# Survival and weak chaos

**DOI:** 10.1098/rsos.172181

**Published:** 2018-05-16

**Authors:** Sean Nee

**Affiliations:** The Braithwaite Group in: Department of Ecosystem Science and Management, The Pennsylvania State University, 410 Forest Resources Building, University Park, PA 16802, USA

**Keywords:** survival analysis, infant mortality, chaos, Pomeau–Manneville map, life-history theory, reliability theory

## Abstract

Survival analysis in biology and reliability theory in engineering concern the dynamical functioning of bio/electro/mechanical units. Here we incorporate effects of chaotic dynamics into the classical theory. Dynamical systems theory now distinguishes strong and weak chaos. Strong chaos generates Type II survivorship curves entirely as a result of the internal operation of the system, without any age-independent, external, random forces of mortality. Weak chaos exhibits (a) intermittency and (b) Type III survivorship, defined as a decreasing *per capita* mortality rate: engineering explicitly defines this pattern of decreasing hazard as ‘infant mortality’. Weak chaos generates two phenomena from the normal functioning of the *same* system. First, infant mortality—*sensu* engineering—without any external explanatory factors, such as manufacturing defects, which is followed by increased average longevity of survivors. Second, sudden failure of units during their normal period of operation, before the onset of age-dependent mortality arising from senescence. The relevance of these phenomena encompasses, for example: no-fault-found failure of electronic devices; high rates of human early spontaneous miscarriage/abortion; runaway pacemakers; sudden cardiac death in young adults; bipolar disorder; and epilepsy.

## 1. Introduction

Ideas of chaos, as formally understood, had a significant impact on many sciences after the invention of computers. One of the first sciences to grasp the significance of such nonlinear dynamical behaviours was population biology, as a result of the work of May [1]. This made it understood that apparently random, and often large, fluctuations in a time series of organism abundance do not require explanations in terms of external perturbations or shocks to the system. Instead, they can be a natural unfolding of the intrinsic, entirely deterministic laws of the system itself.

Here we explore the implications of chaos for the time series of the life and death of the individual organisms, governed by their system design. For example, we will see that many phenomena like sudden, unexplained death that we seek to ascribe to external perturbations, random shocks or simply mistakes will be natural, deterministic and intrinsic to the system itself.

This is the case whether or not the organism's system is designed by an engineer, a Watchmaker—or natural selection, a Blind Watchmaker [2].

The theoretical framework is the classical one of reliability analysis in engineering [3–5] and survival analysis in biology—Cox modelling in particular [6,7]. Reliability engineers have begun the process of uniting these streams under the rubric of failure modelling [8]. We will use both failure and survival theory as general terms and talk about *units* failing, which may be people or machines, for example. We summarize the framework of failure theory in §2 for a combined audience, as each tradition emphasizes certain ideas less known to the other and the idea of failure, as we will see, is more generally useful.

The ideas used from dynamical systems theory will be presented in Models. We use the logistic and the Pomeau–Manneville maps and their associated theory to incorporate chaotic dynamics into survival theory. The logistic is familiar to population biologists, and the fact that it has universal significance has been captured in the phrase the ‘butterfly effect’ for one of its significant features. It is the type specimen for strong chaos.

The Pomeau–Manneville map is well known to mathematicians, and is becoming known in biology for its role in the study of ‘anomalous diffusion’ [9]. The map is the type specimen generating weak chaos. We focus on two features of the dynamics it generates: intermittency, aka sporadic behaviour, and a declining hazard.

Ecology embraced the idea of chaos early, in the context of systems—populations—for which there is: (a) an unavoidable paucity of data and (b) a substantial input of external noise into the unfolding dynamics of the systems.

In this paper, we are discussing the performance of units that are designed to operate in a way that largely insulates them from, or controls, externalities, which will be given a more precise specification in §3.

The evolutionary ecology and reliability engineering of these units will enjoy the benefit of large streams of relevant data for the study of their performance, as we will sketch in the Discussion.

The collection of these data is accelerated by the demands of the industries associated with the evolution of cyber-physical systems including, for example, autonomous vehicles and many such technologies using Artificial Intelligence. Both the enthusiastic public, and the military, now collect data collectively known as the ‘quantified self’, using technologies known to everyone in their simplest forms as Fitbits, for example. These data are naturally collated under the rubric ‘big data’. These data are particularly valuable as they are gathered on healthy systems which are functioning as the designers originally intended, like aircraft carrying black boxes.

## 2. Classical framework

Failure has many meanings [10] including, for example, the end of industrial strikes, as well as more familiar usages such as damaged or deteriorated beyond repair. This may or may not be death, as illustrated by the military use of the word casualty for any failure rendering the soldier out of action for relevant operational timescales.

Failure functions, *F*(*t*), describe the proportion of units that have failed by time *t*, out of a cohort initiated at time zero. ‘Reliability’ is 1 − *F*(*t*), i.e. ‘not failed’ by time *t.* The ‘survival’ function of biology is the same as reliability. So, if you, the reader, are 40 years old, of the people born in the same year as you, a proportion *F*(40) are now dead. If you are a household appliance packaged and waiting to be sold, *F*(expiry date) is related to the proportion of your shelf-mates that will break down before the expiry time limit of the guarantee.

From *F*(*t*) is derived the hazard function, *h*(*t*)—the *per capita* failure rates at time *t* of units that have survived to *t.* So, *h*(40) is your instantaneous hazard rate. In practice, hazards are frequently computed for discrete time intervals like years for humans, so an actuary will describe your probability of dying before you, the reader, reach age 41, and so on.

The average remaining lifetime of units that have not failed by *t* is called the MRL—mean remaining lifetime, aka mean residual lifetime. The MRL is a function of *t,* although rarely explicitly written as MRL(*t*). *F*(*t*), *h*(*t*) and the MRL can each be derived from the other, so failure theory can be developed in terms of whichever is most illuminating for purpose.

To summarize: if you, the reader, are 40 years old, *F*(40) is the fraction of people born the same time as you who are now dead, *h*(40) is the probability you will die before finishing this sentence and MRL(40) is how much longer you can expect to live, calculated from … now.

Units subject to failure we call systems, as they are hierarchical: a system is composed of many components, each of which can itself be a system with its own components. Components can be arranged in a system which can be arbitrarily complex, involving series and parallel designs with hot and cold redundancy [3,11] for example.

The ideas of systems analysis [3–5] make it possible to achieve at least one important simplification in failure theory. Although each component, *i*, in a system can have its own failure function, *F*_i_(*t*), all of these can be mathematically combined with rules based on the design blueprint, to describe a system as having a single failure function, *F*_S_(*t*). This is one justification of the theoretical emphasis given to properties of single *F*_S_(*t*) functions and, from now on, we drop the subscript *S*: we will soon see another important justification. The entire lifetime of a unit is often sensibly modelled as a succession of distinct *F*(*t*) functions.

We now consider the scientific roles of *F*(*t*), *h*(*t*) and the MRL in turn.

*F*(*t*) *per se* plays a role in evolutionary ecology, where 1 − *F*(*t*) defines ‘survivorship curves’ [12]. It is not of great importance in engineering although it *can* play a fundamental role in accelerated failure time models of reliability analysis [4,13], but this is by no means necessary [14,15].

For data representation, the *F*(*t*) function is usually portrayed by Kaplan–Meier plots [16]. For theoretical purposes, the Weibull failure distribution is perhaps the most important one in failure theory with cdf *F*(*t*), and shape parameter *k*:

2.1 $$F(t)=1-\exp(-t^{k}).$$

The hazard function, *h*(*t*), is

2.2 $$h(t)=k{t^{k}}^{-1},$$

with *k* being the shape parameter. Such functions are usually expressed with a scale parameter—is time measured in seconds or years?—but the unit scale is used in this paper to avoid unnecessary symbols. The case of shape parameter *k* = 1 is the exponential distribution and the hazard is a constant, independent of time, with *h*(*t*) = 1. *k* > 1 is increasing hazard—ageing, burnout or senescence in the usual sense. *k* < 1 is decreasing hazard, described as burn-in and infant mortality—the latter term used in engineering with this exact definition.

**Figure 1.**
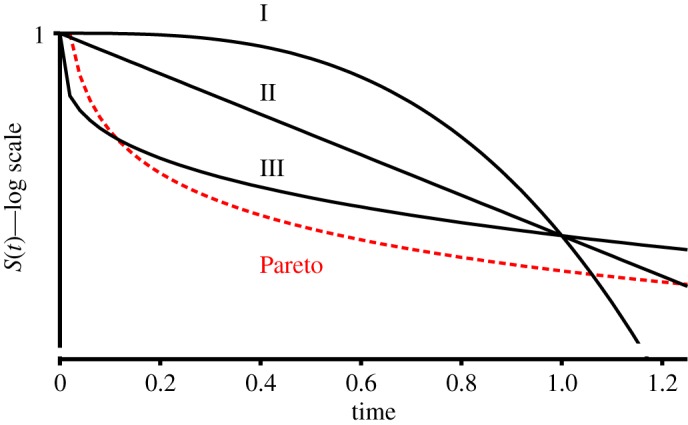
Survivorship curves I, II and III plotted using the Weibull distribution, *k* = 3, 1, 0.3, visually descending. As in ecological practice, *S*(*t*) is on a log scale, allowing the *per capita* survivorship rates of those still alive, and how this may be changing over time, to be visually inferred from the slope. The Pareto distribution—see text—is plotted for *t*_min_ = 0.02, *k* = 0.3.

Recalling that 1 − *F*(*t*) is the survival function, *S*(*t*), ecologists would refer to survivorship curves Types I, II and III for *k* > 1, *k* = 1 and *k* < 1, respectively, although not having the Weibull distribution in mind [12]. These are illustrated in figure 1.

The Pareto distribution is illustrated in figure 1, as it will assume great importance later. It has a particularly simple form:

2.3 $$F(t)=1-{\left(\frac{t}{t_{\min}}\right)}^{-k},$$

for *t* > *t*_min_—the minimum age at which death can occur, here taken to be a very small number for the sake of the figure's appearance. The hazard function, *h*(*t*), is

2.4 $$h(t)\propto \frac{k}{t}.$$

In contrast to *F*(*t*), the hazard, *h*(*t*), is of paramount importance in biology, forming the basis of Cox proportional hazards analysis [6,7] and the comparison of Kaplan–Meier survival curves with log rank tests [16]. The latter is the simplest form of Cox modelling, although this is frequently not explicit in engineering [17,18] as the application of these techniques, to spacecraft reliability for example, is relatively recent in both engineering [19] and biology [20].

The hazard is such an essential quantity of most reliability theory in engineering that it is often simply referred to as the ‘failure rate’ [4].

‘Infant mortality’ is, by definition in engineering, a period of decreasing hazard, so-called because such periods are usually observed early in the lifetimes of units, including high-precision electronic units like semiconductor chips [21], for which the period is, perhaps, surprisingly lengthy.

MRL is a central concept in the understanding of infant mortality and burn-in [22] which, as a theoretical subject, has been deeply studied primarily by engineering [23]. We will see (§4) an important, counterintuitive implication of weak chaos for the study of infant mortality in evolutionary ecology—elevated mortality rates early in the lifespan can be more than made up for by increased longevity in the adult cohort.

The Weibull distribution is not only important for its flexibility. First, it is uniquely valid [10] for both the Cox proportional hazards model [6,24] and accelerated failure rate models [15] of inference. These models are extensively used in applied survival/reliability analysis in both engineering and biology.

Second, the Weibull arises as the distribution of *first* failure times when considering a large ensemble of units [4,25]. This is irrespective of the distributions describing the failure of each individual unit, making the Weibull one of the three ‘extreme value’ distributions—extreme value results achieve generality in a way familiar to us from the genesis of the normal distribution.

Furthermore, the other two extreme value distributions for the random variable time, *T*, are variants of the distribution of ln(*T*), when *T* is Weibull-distributed [26–28]. This form of distribution also arises in Frank's recent work [29] on the foundations of survival theory, again underlining the importance of the distribution.

The importance of the Weibull is well established in reliability engineering [30]. The Weibull and the Pareto are central in the next section—the Weibull in its special, exponential case. Being a power law distribution, the Pareto has attracted an enormous amount of attention over the years under various names [31]. We will see next how it arises for chaotic dynamical systems.

## 3. Models

In this section, we introduce the two simple models the paper is based on—the logistic and the Pomeau–Manneville maps—and identify their implications for our understanding of failure. The models are easily implemented on spreadsheets.

The logistic and Pomeau–Manneville maps exhibit ‘strong’ and ‘weak’ chaos, respectively. Strong chaos naturally provides a deterministic mechanism leading to exponential failure times, even in systems with 100% reliability of their components in the absence of external shocks. Weak chaos generates (a) sporadic failure and (b) Type III survivorship, i.e. failure distributions with increasing hazard. We illustrate these properties and then describe how they are relevant for understanding the failure of units such as organisms.

### 3.1. The logistic map

One important scientific message of the logistic model is that apparently random, often large fluctuations in time series can occur with no random external perturbations, but can be intrinsic to an entirely deterministic system, even an extremely simple one.

Engineers have made this statement more precise. External ‘shock’ models in reliability engineering usually, and reasonably, assume a Poisson process [22,32] for the external occurrences leading to unexpected behaviour, such as accidents, encounters with predators, acts of God etc. This idea has been concisely described by a principal architect of failure theory, Cox [25].

Biologists now well understand that random population fluctuations may have internal deterministic causes, arising from density-dependent population dynamics, and their analyses have reasonably focused on distinguishing internal from external causes, as the latter are so obviously relevant.

They have yet to extend the insight on which this is based to the survivorship of *organisms*. To date, Poisson shock models are more or less explicit in ecologists' discussion of Type II survivorship, i.e. the exponential failure model. The contrast is usually with system failure arising from the senescence, wear and tear, of its components, characterized as *internal* forces and leading to failure models with increasing hazards, which ecologists call Type I survivorship.

This may simply be a result of the lack of an explicit alternative: as we will see, the logistic model itself shows that Type II survivorship can have internal causes, and not be a manifestation of random external shocks. The question of distinguishing such broad alternatives exists in a much more general context, taken up in the Discussion.

So, turning to the logistic map as our starting point, with *x* varying between 0 and 1:

3.1 $$x_{t+1}=rx_{t}(1-x_{t})$$

and we take *r* = 4 throughout.

The behaviour of the logistic is now well understood and has been informative in many fields [33,34], including ecology, to which it was introduced by May [35], where the variable, *x*, is interpreted as population size relative to some maximum. But the model did not originate [36] as a cartoon of animal multiplication, and its scientifically wide-ranging importance comes from the fact that discrete time maps like the logistic, have a close, albeit imperfect [37] relationship to Poincaré maps of higher-dimensional, continuous time processes and, hence, can illustrate the major features of the dynamical behaviour of such systems.

**Figure 2.**
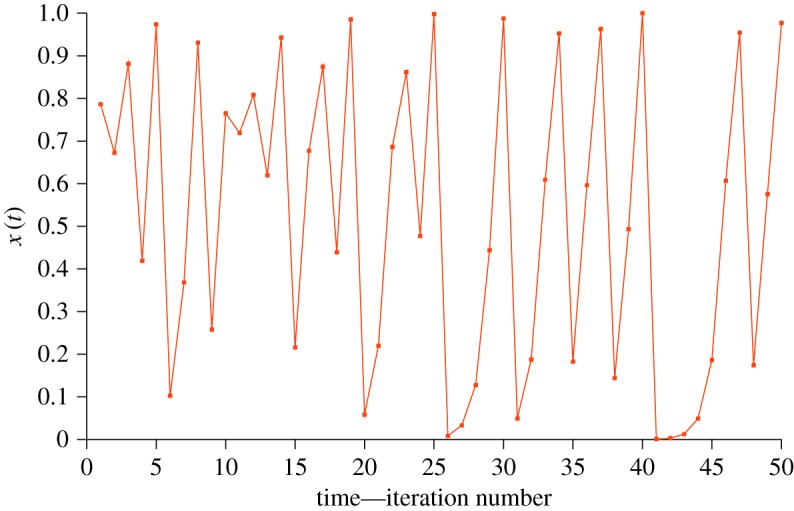
The map equation (3.1), with *r* = 4. This map was originally suggested by John von Neumann as a random number generator for use in the first computer programs [36].

Iterating this map generates a times series, *x*(*t*), figure 2, with apparently random—chaotic—dynamics:

Two maps closely related to the logistic are the tent map and the dyadic, aka Bernoulli:

The tent map is

3.2 $$\begin{array}{rl} x_{t+1} & =2x_{t},\;0<x<0.5\\ & =2-2x_{t},\;0.5<x\leq 1. \end{array}$$

The Bernoulli map:

3.3 $$\begin{array}{rl} x_{t+1} & =2x_{t},\;0<x<0.5\\ & =2x_{t}-1,\;0.5<x\leq 1. \end{array}$$

This can be written in terms of modular arithmetic as

3.4 $$x_{t+1}=(2x_{t})\mathrm{mod}1.$$

**Figure 3.**
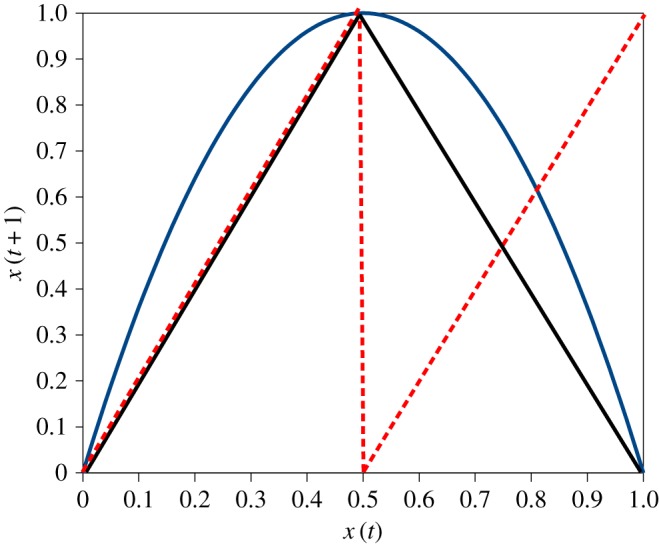
The logistic map, *r* = 4, is the parabola. The tent map, solid black, appears as a straightened version of the logistic, and the Bernoulli map as a broken tent map, in dashed red. We retain the graph wall to create an image of a box for a particle to bounce around in, according to the map rules.

All three maps are illustrated in figure 3.

We can imagine a ball bouncing around in this unit square box, figure 3, according to the map rule and the successive values {…*x*_*t*_, *x*_*t*+1_, *x*_*t*+2_ …} being recorded as the time series, figure 2. Mathematically, these three maps are conjugate, which means they have the same dynamical features—in particular, they exhibit chaotic dynamics.

The maps differ in two notable respects. First, the tent and the Bernoulli maps are very useful for mathematicians for reasons suggested by their simple, linear forms: but they function as ‘bit-shift’ maps, which renders them useless to us for study by normal simulation, which quickly runs afoul of the limited precision of the representation of numbers in computers—simulation on a spreadsheet will make this clear.

Second, the Bernoulli map is essentially identical to the logistic in terms of its relevant behaviour, but has no imaginable interpretation in terms of population biology. This underlines the fact that the scientific importance of the logistic does not lie in its presentation as a cartoon of temperate insect infestations, although this is often didactically useful.

It is additionally useful to us here as it naturally leads to the Pomeau–Manneville map.

### 3.2. The Pomeau–Manneville map

The modular form of the Bernoulli map, equation (3.4), is easily implemented using the MOD functions on spreadsheets. In this form, it is readily generalized to

3.5 $$x_{t+1}=(x_{t}+ax_{t}^{z})\;\mathrm{mod}1.$$

The Bernoulli map is a special case of this with the parameters *a* = *z* = 1.

**Figure 4.**
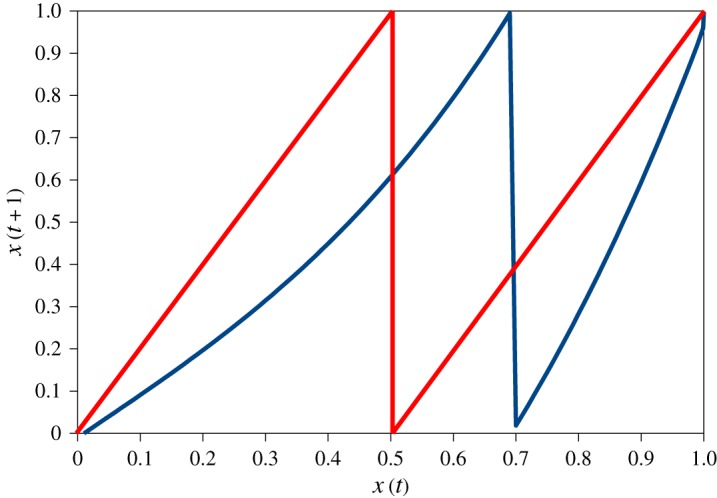
More maps in a box: Bernoulli map (red) and the Pomeau–Manneville map (blue).

The general map of equation (3.5) is called the Pomeau–Manneville map, and for ready comparison with the case treated by Klages [9], a principal source for this section, we take *a* = 1 and *z* = 3 throughout. This map is illustrated in figure 4.

**Figure 5.**
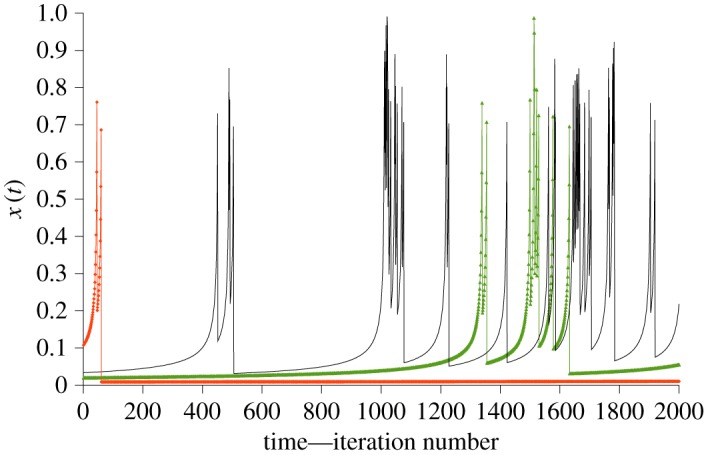
Three typical time trajectories of the Pomeau–Manneville map with initial conditions close to zero and parameters as described in the text. Repeated simulation suggests that no run ‘takes off’ before about 40 iterations.

In figure 5, we see the typical behaviour of the map for three different values of *x*_0_ starting at random points in the interval (0.01, 0.11).

The principal, obvious feature of this behaviour is intermittency, in which periods of stability are punctuated at intervals by chaotic bursts. This is the characteristic of the Pomeau–Manneville map which is of most interest here.

In the gestation of the study of nonlinear dynamics, intermittency behaviour has been seen as describing a thin transitional layer in between zones of regular periodic and strongly chaotic behaviour [38].

The straightforward graphical generation of the Pomeau–Manneville map makes it clear that models exhibiting intermittency do not reside in a bizarre backwater of the mathematical imagination, a peculiar thin layer of parameter space. Gaspard & Wang [39] says the sporadic behaviour illuminated by the Pomeau–Manneville map ‘fills in a gap … between predictable and random patterns': between black and white, there is much grey.

The Pomeau–Manneville map describes a dynamical system moving between different regions of dynamical space, in this case a particularly simple space with smooth and chaotic regions, as seen in figure 5. In one region, we see the chaotic dynamics characteristic of the Bernoulli map in which large, unpatterned spiking can occur, whereas the other has smooth, laminar flow: the movement between these regions generates the intermittency.

In this particularly simple case, the regions are defined in terms of a reasonably stable, predictable output being interrupted by unstable, unpredictable output of indeterminate length with, importantly, no external perturbations of any sort. Hence, the system has a mind of its own.

The map is said to exhibit ‘weak’ chaos, in contrast to the ‘strong’ chaos of the logistic map, with the following meaning.

The logistic map exhibits sensitivity to initial conditions throughout, a phenomenon popularly called the butterfly effect. Specifically, two trajectories that start very close to each other will exponentially diverge and ‘forget’ their initial proximity, and this is quantified by the Lyapunov exponent of the map.

This is most immediately seen by reference to the Bernoulli map, equation (3.3), in which two trajectories that are a very small distance apart, *Δx*, at a time *t*, will see their distance apart, *Δx*, grow through *m* successive iterations as

3.6 $${\left(\Delta x\right)}_{m}=\Delta x2^{m}=\Delta x\exp^{m\ln2},$$

i.e. exponentially at a rate ln2—this rate is called the Lyapunov exponent. This is the same Lyapunov exponent of the conjugates—the tent map and, less obviously, for the logistic map. The definition of chaos in terms of a positive Lyapunov exponent is popular in scientific usage, but is not the only one [40].

In contrast, the Pomeau–Manneville map has a Lyapunov exponent of zero. Intuitively, looking at figure 5, this is a consequence of the fact that two trajectories that are both close to zero at a particular time will diverge very slowly from each other, until they encounter a chaotic zone after which their futures diverge dramatically.

We will discuss specific examples of intermittency in the next section, arising in such natural places to look as cardiac and brain systems.

### 3.3. Survival/failure from the maps

We now describe the derivation of the distribution of failure times of units whose entirely deterministic time trajectories are generated by these maps. So we describe the contemporary theory that explicitly bridges the gap between the deterministic behaviour of individual units with the statistical description of ensembles of such units. None of this is mathematically novel [40], although it is contemporary and novel in this scientific context.

The behaviour of the strongly chaotic maps—logistic, tent and Bernoulli—is as follows. They have a well-behaved ‘invariant probability density’ on (0,1), which we will denote by p*(*x*), which is understood as follows. A particular trajectory {… *x*_*t*_, *x*_*t*+1_, ….} will, over long periods of time, find itself in infinitesimal pieces, d*x*, of (0,1) with an overall frequency p*(*x*)d*x*. An ensemble—a large number of trajectories with different start points spread over (0,1) with density p*(*x*)—will be mapped back into (0,1) with the same density at each iteration of the map.

This ergodic property—in which the time and space average behaviour is the same—is what links the behaviour of an individual trajectory when viewed over a long period of time to the average behaviour of a large number of trajectories over short periods, which provides us with the statistical framework we need here.

For the tent and the Bernoulli map, the density p*(*x*) is the uniform density, a constant = 1, whereas for the logistic map

3.7 $$p^{*}(x)\propto x^{-1/2}(1-x^{-1/2}).$$

This latter accounts for the apparent concentration of points in the extremes in figure 2. Use of the tent or Bernoulli map would, in principle, provide a more familiar random scatter over (0,1) but, as noted before, this is not easily done with the limited finite representations of numbers in simple spreadsheet simulations.

To model failure, we can define a death region so that any trajectory entering the region ‘dies’—is terminated. In simulation practice with the logistic map, for example, one can simply define a threshold, *x*_f_, above which failure occurs. Although this procedure uses entry into the extremes to decide when failure occurs, we could use any small interval in (0,1) we like.

By choosing any specific region of small size, d*x*, the proportion of a population that is mapped into this on each iteration and, hence, fails will be geometric with parameter p*(*x*)d*x*. If this probability-per-iteration is small, say *μ*, we can pass to a continuous time description and find that *S*(*t*), the proportion surviving as a function of continuous time, is exponential:

3.8 $$S(t)=\exp^{-\mu t},$$

the ecologists’ Type II survival curve of slope *μ*.

We turn now to the behaviour of the Pomeau–Manneville map, which can be understood through its mathematical definition, equation (3.5), and sample trajectories, figure 5, as follows. There is an equilibrium at *x* = 0 which is: *marginal* aka *indifferent*, meaning that trajectories in its vicinity are *sticky* and the system moves very slowly away from it [9]. The initial values in figure 5 were chosen to be close to this equilibrium. Away from this region of smooth, *laminar* flow, there is a region of chaotic dynamics akin to that of the Bernoulli map.

Coexistence of these regions results in trajectories that begin close to zero moving slowly away until they reach the pull of the chaotic region. This happens near the 40th iteration for the parameters chosen for this simulation. Then follows a wild chaotic ride, before the system is flung back to a random location in the tranquillity near zero at a random injection point.

So, we have the idea of two regions, one consisting of smooth, laminar flow and the other consisting of the sort of chaos we are accustomed to from the chaotic maps which injects points randomly back into the laminar region. Assigning the locations of these injection points a uniform distribution allows one to study the trajectories over time generated by the interaction of these two regimes [41].

As with the logistic map, here we define the failure of a unit to occur when its trajectory enters an extreme region, e.g. *x_t_* > 0.9 in figure 5. This will only happen when the trajectory enters the chaotic zone. The closer a trajectory is injected to the indifferent point at zero, the longer the time taken to increase again into the chaotic zone. This length of time increases to infinity as proximity to zero becomes infinitesimal, so this time distribution governs the dynamics generating the failure distribution.

The basic result is that the distribution of failure times is of the power law form, involving time as *t*^−α^ [39,41], whose most famous incarnation is the Pareto distribution, which we saw in figure 1 and equation (2.3).

So, weak and strong chaos are readily incorporated into survival/failure theory mathematically. Now, we consider the scientific implications. In particular: first, we consider the consequences of weak and strong dynamics in the absence of any change in the operating environment and, second, consider also the effects of such changes. The implications will be presented in stark simplicity in this section with vivid examples for exposition purposes.

Cardiac systems are an obvious place to look for interesting nonlinear phenomena [42–44]. The ECG records of patients experiencing ‘runaway pacemakers’, which have resulted in patient ‘failure’, look remarkably like figure 5, characterized by a normal, rhythmic pattern interrupted by periods of fast, erratic spikes [45]. This phenomenon is only observed well into the operational lifetime of the pacemaker and, in the absence of alternative explanations, is ascribed to battery rundown. The idea that the passage of time itself is the relevant temporal correlate is not entertained, and the runaway phenomenon is also observed with newer technology [46].

The sudden cardiac death of young adults which is classified as unexplained is about 30% [47]. Unlike the battery ageing explanation of runaway pacemakers, there are no obvious senescence explanations for sudden cardiac death. Hypothesized explanations usually invoke undiagnosed abnormalities. Weak chaos tells us that it is entirely possible that failure has occurred as an unwanted outcome of normal operation. As this dynamical possibility alone is unlikely to make any more useful contributions to this subject than previous invocations of other nonlinear phenomena, how one might proceed to make a more useful contribution will be taken up in the Discussion.

The brain is a spectacularly nonlinear dynamical system fraught with chaos [48], and intermittency phenomena are well known, such as with epilepsy [49] and, with less abrupt transitions, bipolar disorder [50]. This will be taken up in the Discussion. Fortunately, these particular manifestations of weak chaos do not often take the system entirely outside its survivability region.

Strong chaos is also readily incorporated into the failure theory of engineered systems when we recall that chaos does not mean unpredictability—series of coin tosses are memoryless, dynamically chaotic systems with highly predictable behaviour, and the logistic map is mathematically equivalent to such a series. From the beginnings of the study of Chua's circuit, the development of chaotic computer chips and ‘chaos computing’ [51] has developed in an explicitly nonlinear dynamical framework.

A system may be designed with a chaotic component whose failure function is perfectly acceptable for use, even though it will exceed performance tolerance limits at a time that is unpredictable individually but statistically entirely predictable and acceptable.

Hence, a clear result from the model of strong chaos is that Type II survivorship—an exponential failure distribution—can arise without any external causes or background of external shocks. Nor does it require any invocation of the degeneration—senescence—of components.

Both sorts of chaos are relevant for understanding the failure of equipment explicitly described in engineering as resulting from a No-Fault-Found failure mode [52], a common phenomenon. Note that we are not suggesting chaos as a Ghost-in-the-Device, a GID of the explanatory gaps, however—see Discussion.

We now briefly consider the potential qualitative effects of *changes* in the operating environment of a unit by considering changes in the parameters of the simple models.

The behaviour of the logistic map is the canonical example for the period-doubling route to chaos [33]. As *r* in equation (3.1) increases from 1, the system behaviour goes from exhibiting a single stable equilibrium, to 2 cycles, to 4 cycles, 8 cycles etc. until chaotic regions start to be encountered—we studied the chaotic region encountered at *r* = 4.

Consider a system operating at a stable attractor, some *r* < 4, which may be a point or have an oscillatory nature. In this case, the system has 100% reliability in the absence of external shocks. However, a small change in the operating environment—abstractly captured in the parameter *r*—can transform the system's dynamics to a form exhibiting exponential failure.

This has two implications for the scientific understanding of failure. First, startling, anomalous phenomena may not require *shocking* causes, in the technical as well as colloquial senses, but can arise from a smooth alteration of the operating environment. Second, it points to a direction for giving the theory an applied dimension—taken up in the Discussion.

For weak chaos, experimenting with the parameter *z* of the Pomeau–Manneville map, equation (3.5), shows that the threshold moves, the threshold being where the pull of the chaotic region overpowers the indifference of the equilibrium at zero. So, changing the operating environment of a system may move the regions around, and continued performance stability may disguise the fact that a very different pattern of dynamical behaviour has moved closer in dynamical space and operating time.

Such behaviour in more advanced forms is vividly illustrated by the behaviour of two or more linked pendulums. An example is provided by Rott's pendulum: we see on YouTube—e.g. between 1:00 and 1:28 in [53]—oscillatory systems with a region of stable/regular output surrounded by regions with very different properties, and a rapid transition between them. Such systems are both known and extremely common in biological and non-biological electronic systems.

In the case of sudden cardiac death in young athletes, obvious novel changes in the operating environment of a young adult's cardiac system, are unusual kinds of muscle development, nutrition, energy expenditures and hormones. Young adults are prone to the onset of other unwanted anomalies in the functioning of their highly nonlinear systems—like schizophrenia. Again, the Discussion takes up how to transition from musings to useful knowledge.

Generally speaking, theory tells us that for shocking events, we may have to look inside the normal functioning of the system, as such events may, in fact, be perfectly natural and a consequence of the design. In addition to what we have learned from strong chaos, weak chaos shows us that: (a) the system can also move between qualitatively different behaviours ‘of its own accord’, i.e. without any external changes at all; (b) changes in operating environment can move the dynamical zones around relative to one another, changing the timescales on which the system moves between them.

The view of intermittency we promote in this paper is emphasizing the existence of different regions of an abstract dynamical space, defined by distinct, discernable patterns of behaviour, which a system can move between. Engineers intentionally create the circumstances of such phenomena in the practice of burn-in, which we take up in the next section, where we suggest that also it may be observed occurring naturally.

## 4. Infant mortality and burn-in

We recall that the mean remaining—residual—lifetime of a unit is a function of time, *t*; it is the average additional period of time a unit will survive, given that it has already survived to *t*.

The essential result from modelling, intuitive but not straightforward to prove [54], is that distributions with a monotonically increasing hazard have a decreasing MRL, and vice versa. So, 40-year-old readers of this article have a higher hazard and shorter MRL than 30-year-old readers.

This fact has a rich understanding in terms of the evolutionary theory of senescence, as described by Hamilton [55]. That framework understands senescence in terms of the action of genes which have fitness benefits earlier in life, but exact a cost later. Hamilton clearly stated the difficulty of understanding infant mortality in that adaptive framework and suggested additional ideas, such as the loss of some offspring being of benefit to survivors.

What we will see here is that system dynamics can naturally produce a cohort of units which will exhibit infant mortality and increased longevity of the survivors.

This phenomenon is seen in the MRL of units with *decreasing* hazards, so the longer you have already waited for an event such as death, the more additional time you can *expect* to wait.

We remind ourselves that infant mortality is the phrase used in engineering, and here, to describe an early period in the lifetime of units exhibiting a declining hazard. It is ubiquitously observed early in the lifetime even of units manufactured with very high precision, like semiconductor chips [21]. It is also striking in humans, where early spontaneous abortion results in at least 30% of initiations failing [56].

In engineering, the phenomenon of infant mortality is usually attributed to manufacturing defects, considered to have failed to be eliminated in even high precision manufacture. In biology, the same reasoning invokes unknown genetic causes as an explanation [57], on the supposition that they exist, albeit currently unidentified. There are many cost-and-benefit desiderata that are analysed deeply by engineers, such as the increased manufacturing costs required to reduce early infant mortality rates.

We will see here that infant mortality is a natural occurrence in systems whose functioning exhibits features of weak chaos and that a statistical effect of such mortality is the increased life expectancy of the survivors.

The engineering term burn-in is a useful one, as it allows a distinction between two time periods. The first begins at inception, and then a later time when the unit is put into operation—or marketed—if it has survived. The time in between is the potential burn-in period. This may be, for example, the time between a unit coming off the assembly line and arriving, packaged, for sale in a store if it has survived, or the time between the fertilization of an egg and birth, and so on.

The phrase burn-in arises because, in engineering, efforts are made to accelerate and weed out supposedly defective units before marketing, and the term burn-in usually refers to such interference [22,23]. In biology, it is assumed to happen naturally. A notable exception is the practice of the military to weed out recruits. In fact, we are used to this idea in the manufacture of soldiers—recruits are mistreated during boot camp to weed out recruits that are unlikely to last long as soldiers.

We are not here wedded to the fact that engineers, or the military, typically interfere to *increase* failure during the burn-in period. There is much engineering literature concerning optimal management of burn-in periods and the modelling does not mathematically require efforts made to *accelerate* failure during the period. By discarding this assumption, we also discard the implicit assumption made for the manufacture of organic units that efforts are made to *reduce* failure, if anything.

So, we use our simple models to view the potential of the increased MRL of those units that survive and are marketed after a time *t* to compensate for the operating time lost by those units that fail before time *t*. In these models, as before, failure is an entirely intrinsic, deterministic outcome of the natural dynamics of the system.

To proceed, we want to see how simulations behave, as this is the information available to engineers and Blind Watchmakers, not mathematical results concerning processes over infinite timescales, for example. We do have some knowledge of the latter, which we can use to help understand the former.

We model the case of *no* interference—the product is simply run in the factory before being sold, for a period of time we call the burn-in period. We run simulations of 100 units obeying a specified model and ask how the length of the burn-in period, *t*, affects the MRL, calculated from *t*, the end of the burn-in period. We run the simulations for a total of 2000 time units. Our parameter choices are such as to be consistent with our previous discussions.

In the results we report, there is clearly censorship—we do not know how long survivors would last beyond the timescale of the study. There are autocorrelations in the MRL as a function of burn-in period for individual runs, and so on. As we are describing illustrative simulated data, we must simply be aware of these facts in order to understand what we are seeing, rather than attempt to statistically magic them away simply out of habit. But more importantly, these effects will be relevant to the engineering decisions of watchmakers, blind or sighted, even though they may be distracting when seeking mathematical insights into infinity.

**Figure 6.**
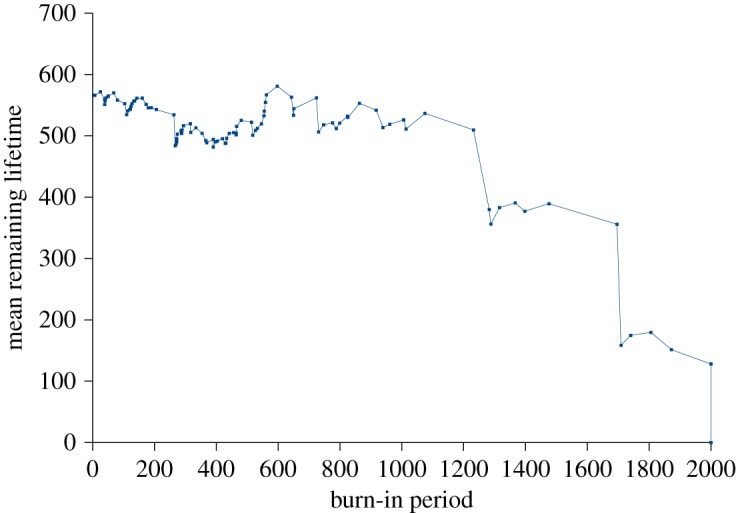
Mean remaining lifetime function of a typical run of the logistic map as previously described. Here, and throughout, the points are plotted at each failure event, as this information may also be informative to the reader.

A typical simulation of the MRL of an ensemble of units, each with a trajectory determined by the logistic model, is illustrated in figure 6.

These simulation results from an explicit deterministic model accord with the theoretical understanding. For the exponential distribution, equation (3.8), it is clear that the MRL, the time to failure conditional upon failure not having occurred by time *t*, is a constant. This ‘memoryless’, ageless property is central to the interpretation of exponential failure in ecology—Type II survival—as resulting from random external factors independent of the age of the units, and is the basis of many popular probability ‘paradoxes’.

As expected, the simulated MRL is flat until censorship starts to lower it—simulation readily reveals that any imagined patterns in the early ‘flatness’ are an illusion.

**Figure 7.**
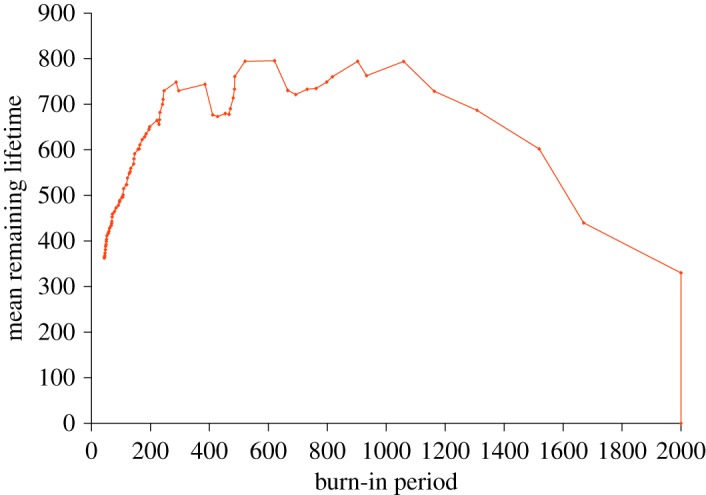
The MRL of a typical simulation of the Pomeau–Manneville map, with parameters as described in the text, grows rapidly.

The MRL of a typical simulation of the Pomeau–Manneville map (figure 7) is increasing. As before, this simulation must be viewed in awareness of the existence of censorship and autocorrelation.

The growth of the MRL is expected. Theory discussed in §3.3 tells us that the Pomeau–Manneville map leads to a failure time distribution of the Pareto form, and further informs us [58,59] that the shape of the Pareto distribution, *k* of equation (2.3), is related to the Pomeau–Manneville map parameter, *z*, as *k* = 1*/*(*z − *1). The MRL of the Pareto distribution, is *t*/(*k − *1), *k* > 1, which informs us that the mean and the entire MRL are mathematically infinite. Scientifically, this infinite behaviour makes power laws like the Pareto widely used in the context of long-tailed distributions, like those including Bill Gates' fortune and spacecraft lifetimes as long as that of Voyager 1, which is still working as of December 2017 as it leaves the solar system.

Just as much infant mortality in engineering *does* obviously result from manufacturing errors, a high percentage of the early spontaneous abortion rate in early human pregnancy has identifiable causes in terms of chromosomal abnormalities [57]. This will be reduced to the extent that the manufacturing technology is available to do so and is cost-effective. What we now see is that, from a designer's point of view, nonlinear systems with desirable features may also exhibit infant mortality and increased MRL as a combined package.

We have discussed the behaviour of the Pomeau–Manneville map in terms of the system moving between different regions of behaviour in a dynamical space envisaged in any way that is appropriate. A related viewpoint is also pertinent. Mathematicians study models of diffusion in which the observable phenomena of, for example, Brownian motion are generated by explicitly deterministic models at the micro level. This is modelled, in effect, by connecting boxes such as those in figures 3 and 4 and allowing the bouncing particles to escape through holes from one box to another [9].

Because of the sticky equilibrium of the Pomeau–Manneville map, models of diffusion using it exhibit what the mathematicians call ‘ageing’, as particles tend to accumulate in the vicinity of such equilibria [59]. This is an unfortunate term for us as scientists as it has many too many associations with quite different phenomena.

## 5. Discussion

The incorporation of nonlinear dynamical ideas into population biology was very rapid after May [1,35] dropped the flag. A striking example of what we would today call intermittency was long recorded in the outbreaks of insect pests such as spruce budworm, which exist for long periods at low densities before intermittently spiking to great abundance. A qualitative dynamical analysis [60] of the phenomenon invoked many ideas we would now associate with weak chaos, such as different relevant timescales with sketchings and stretchings of state space with both potentially indifferent equilibria and unstable equilibria at the centre of potentially chaotic regions. The rich webs of ecological relationships between populations readily generate interesting dynamical phenomena in ecology.

This is both a blessing and a curse, as it means population biology is naturally deficient in a supply of useful datasets, required for pertinent analyses. This cannot be remedied, but there are theoretical advances still to come that will assist analysing such data that we have. For example, early studies looked for the signature of chaos in Lyapunov exponents [61], which we now see is no longer a gold standard when analysing time series.

Theory also serves to point in fruitful directions to look for internal mechanisms known to destabilize population dynamics, such as the over-compensating mode of density-dependent mortality [62].

Engineers have explicitly discussed such alternative, and complementary approaches of the statistical approaches and the mechanistic study of the ‘physics-of-failure’ in publications for governments, as the abundance of, for example, nuclear reactors is large enough for this to be meaningful [63,64]. In addition, the fact that ideas from applied mathematics must be incorporated into statistical inference has been made in a non-trivial manner by Cox in his chapters on probabilistic models of failure [25] and modes of machine interference in queuing theory [65]. Biologists are joining this tradition of discourse.

To proceed from here, when considering systems such as organisms, machines and computer programs, we can start from the premise that they are nonlinear systems manifesting a potentially vast range of behaviours which we do not actually understand. Then the challenge is to develop this understanding of their operation, rather than puzzle extensively over the relative importance of internal versus external causes of their behaviour, as we have been forced to do with population biology.

A strategy recently suggested by, in fact, population biologists is to explore the dynamical space in which system behaviour lives to find ways to detect that a system is en route between dynamical behaviours [66]. Such an approach has been previously suggested explicitly in the context of cardiology [67,68].

These examples look to data from carefully studied populations or organisms under stress or in distress, such as endangered species, premature infants or heart patients in intensive care units. The future lies in the tsunami of data to be forthcoming from the functioning of systems behaving normally. This occurred in the airline industry by commercial necessity, which introduced black box recorders into all aircraft after several mysterious disasters. It is occurring naturally now in many new areas, with some examples below.

The proliferation of sophisticated electronic devices and soon cyber-physical systems like driverless cars will provide much data. There is pessimism, it so happens, about the accumulation of sufficient data for statistical reliability analysis of the performance of autonomous vehicles [69], but the fact that engineers are even thinking explicitly about this outlines the shape of things to come.

The military has been a voracious consumer of reliability theory and data for decades, published in its Military Handbooks MIL-HBK [70], and is now explicitly extending to the performance of its human units [71,72] these techniques better known as survival analysis to biologists. It has vast funds to gather data from healthy, young individuals, a proven source of information for such militarily irrelevant topics as the evolutionary ecology of microbes [73].

The healthy public is now collecting its own cloud-connected data with such devices as Fitbits and technologies to continuously monitor various aspects of infant functioning – a crowd enterprise called the quantified self [74].

We may soon see a fusion of the disciplines of engineering and psychology. Engineers are constructing Artificial Intelligence systems operating and self-advancing with Deep Learning; the military is studying the mental health of its soldiers; and biologists have intense interest in the nonlinear modelling of phenomena such as bipolar disorder [50], a disorder characterized by periods of relative stability interrupted by extreme fluctuations of affect—an excellent description of intermittency.

Epilepsy is an even more striking illustration of intermittency, almost by definition. A recent study [49] presents a remarkable synthesis of: (*a*) the analysis of ‘big data’ sets; (*b*) using statistical techniques informed by (*c*) an understanding of nonlinear dynamics. This is the modern world.

## Data accessibility

This article has no additional data.
